# Mitigating cognitive decline in Alzheimer's disease dementia by enhancing cognitive reserve through neuroplasticity in addition to amyloid-β reduction

**DOI:** 10.3389/fnagi.2026.1769431

**Published:** 2026-03-30

**Authors:** Takayoshi Ubuka, Kazuko Yuyama, Akito Genjima, Mika Nagakura, Keiko Kato, Sayuri Hayashi, Naofumi Makino, Takeshi Yamane, Takeshi Hirose, Yoichi Yamane

**Affiliations:** 1 Department of Healthcare, SI Research Institute, SI Holdings PLC., Chuo City, Japan; 2 SI Research Institute, SI Holdings PLC., HOSO-Kiko General Incorporated Association, Chuo City, Japan

**Keywords:** Alzheimer's disease dementia, amyloid-β antibody treatment, cognitive reserve, neuroplasticity, non-pharmacological intervention

## Introduction

Despite recent advances in amyloid-β–targeting therapies, cognitive decline in Alzheimer's disease (AD) dementia remains insufficiently delayed or mitigated, suggesting that reducing pathological burden alone may be insufficient to substantially alter the clinical trajectory of the disease. In this Opinion, we argue that non-pharmacological interventions should be understood as strategies to enhance cognitive reserve (CR) through neuroplasticity, rather than as adjunctive symptomatic treatments of AD.

This Opinion builds upon our previous work highlighting the importance of non-pharmacological interventions for enhancing CR in Alzheimer's disease (Maki et al., 2024), but extends it in several important ways. First, we place a stronger emphasis on neuroplasticity as the biological substrate of CR, integrating human neuroimaging evidence with emerging mechanistic data from preclinical models of AD. Second, we focus on network-level changes, particularly functional connectivity, as a unifying framework linking cognitive stimulation to resilience against pathology (Stern et al., 2020; Park and Bischof, 2013; Nyberg et al., 2003). Third, we use cognitive stimulation therapy (CST) as a translational example to illustrate how non-pharmacological interventions may modulate brain networks rather than merely provide symptomatic benefit.

CR reflects the capacity of neural networks to maintain cognitive function despite AD pathology. Epidemiological, clinical, and neuroimaging evidence indicates that cognitively stimulating activities and social engagement delay the onset of clinical dementia. Among available interventions, CST represents a clinically feasible example, with emerging evidence of increased functional connectivity in brain networks related to memory and sensorimotor integration.

We propose a conceptual model contrasting pharmacological approaches that aim to reduce AD pathology with non-pharmacological approaches that increase tolerance to pathology by enhancing neuroplasticity, thereby delaying or mitigating the clinical expression of cognitive decline rather than fully reversing pathology itself. This perspective highlights the need to reposition non-pharmacological interventions as core components of AD dementia resilience-enhancing strategies rather than optional complements to drug therapy of AD.

## Limitations of pathology-centered treatment

Dementia is a complex condition involving the interplay of various molecular pathways, leading to disruptions in functional networks underlying cognition, personality, behavior, and sensorimotor functions. Aging is the most significant risk factor for dementia. Despite advances in amyloid-β antibody treatments, AD dementia remains incurable, in part because age-related changes in cognitive and neural systems are unlikely to be fully reversible through amyloid-β-targeting agents alone (van Dyck et al., 2023). Although cholinesterase inhibitors are primarily used to treat cognitive symptoms of AD dementia, these drugs have a poor risk-benefit relationship indicated by frequent discontinuation (Blanco-Silvente et al., 2017; Maki et al., 2024).

## Cognitive reserve explains clinicopathological dissociation

Given the inevitability of aging, it is worthwhile to reconsider approaches to modifiable factors. One promising area is the re-evaluation of non-pharmacological interventions aimed at enhancing CR (Maki et al., 2024). CR refers to “the adaptability (i.e., efficiency, capacity, flexibility) of cognitive processes that helps to explain differential susceptibility of cognitive abilities or day-to-day function to brain aging, pathology, or insult” (Stern et al., 2020). To utilize non-pharmacological interventions in dementia therapy, it is important to characterize the neural mechanism of non-pharmacological interventions compared with that of pharmacological interventions.

A landmark study that began in 1986, known as the Nun Study, revealed that some individuals who had been cognitively and physically intact had significant AD neuropathology and vascular lesions at autopsy (Snowdon, 2003). Following studies suggested that subjects with educational level of a bachelor's degree or higher and/or with higher linguistic density (e.g., complexity, vivacity, fluency) in their autobiographical essays were less likely to develop AD in their later life (Riley et al., 2005). It was also suggested that engagement in some sort of daily exercise decreases the risk of developing AD. Following epidemiological studies established that lifetime exposures, such as education, occupation, and leisure activities in late life decreases the risk of developing dementia, possibly by increasing CR (Stern, 2012). Age, gender (female), and low education were highly significant and independent risk factors for dementia in a probability sample survey of noninstitutionalized older persons in Shanghai, China (Zhang et al., 1990).

A cohort incident study showed that the risk of dementia was increased in subjects with either low education or low lifetime occupational attainment (Stern et al., 1994). Risk was the greatest for subjects with both low education and low life-time occupational attainment (Stern et al., 1994). Another cohort incidence study showed that the risk of dementia was decreased in subjects with high leisure activities, such as knitting, music, walking, visiting friends or relatives, being visited by relatives or friends, physical conditioning, going to movies or restaurants or sporting events, reading magazines or newspapers or books, watching television or listening to the radio, doing unpaid community volunteer work, playing cards or games or bingo, going to a club or center, going to classes, and going to church or synagogue or temple (Scarmeas et al., 2001).

**Figure 1:**
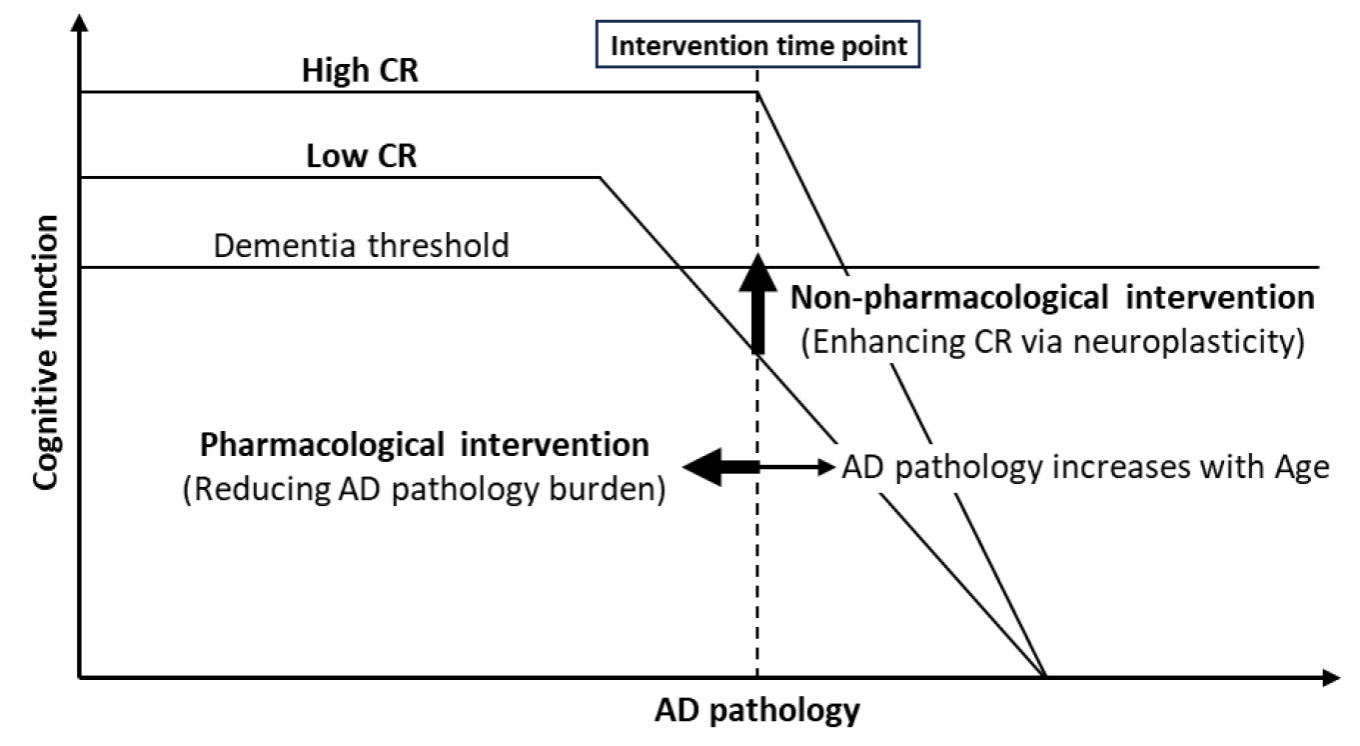
Conceptual model contrasting pharmacological and non-pharmacological interventions for preventing or mitigating cognitive decline in Alzheimer's disease (AD) dementia. This conceptual illustration is adapted from prior cognitive reserve models proposed by Stern (2012) and Stern et al. (2020), with modifications to incorporate pharmacological and non-pharmacological intervention strategies. Amyloid-β pathology accumulates with age and precedes the onset of clinical dementia. Individuals with higher cognitive reserve (CR) can tolerate greater pathological burden before crossing the dementia threshold. Pharmacological approaches aim to reduce AD pathology, whereas non-pharmacological interventions enhance CR through neuroplasticity, thereby increasing tolerance to pathology rather than directly reducing it.

Although cerebral amyloid-β aggregation is an early pathological event in AD, it starts decades before the onset of dementia and increases until death (Jansen et al., 2015). The lower incidence of clinically manifest AD in individuals with higher CR suggests that cognitive decline tends to occur later compared to individuals with lower CR. Magnetic resonance imaging (MRI) identified that mild cognitive impairment (MCI) patients with higher education as a proxy of CR remained cognitively stable for longer years before converting to AD, even if they had similar levels of cortical thinning (Querbes et al., 2009). However, more rapid decline in cognitive function was observed in higher education attained AD patients, possibly due to harboring/tolerating a higher pathological burden at the time of clinical dementia for subjects with higher education (Scarmeas et al., 2006). These results suggest that individuals with higher CR may tolerate a greater pathological burden before the onset of clinical symptoms, followed by a more rapid decline once compensatory mechanisms are exhausted (Stern, 2012) (Figure 1).

## Neuroplasticity as the biological basis of cognitive reserve

As amyloid-β antibody treatments are aimed to decrease AD pathology in the brain, its possible mechanism to delay cognitive decline may be to reverse the accumulation of amyloid-β that may increase until death without treatment (Jansen et al., 2015) (Figure 1). On the other hand, non-pharmacological approaches may mitigate cognitive decline by increasing CR of the people in the presence of AD pathology (Figure 1). It is therefore important to identify the neural mechanisms by which non-pharmacological interventions may enhance CR through neuroplastic changes at the structural, functional, and network levels (Figure 1).

The aging brain must have the capacity of neuroplasticity that is the ability to change structure or function in a sustained manner in response to external stimulation to improve cognitive function (Park and Bischof, 2013). The most compelling evidence of neuroplasticity of older adults comes from stroke patients who had permanent damage to their brain in specific areas due to neural bleed or blood clot. Stroke patients can show dramatic recovery through many hours of intense therapy to regain function (Ramanathan et al., 2006). It was demonstrated that aerobic exercise can delay shrinkage in prefrontal cortex in sedentary older adults (Colcombe et al., 2006). There are also reports showing that cognitive interventions can increase neural volume in hippocampus and nucleus accumbens in older adults (Boyke et al., 2008; Lövdén et al., 2012). There are also considerable body of literature showing that cognitive interventions can enhance neural activity in older adults (Nyberg et al., 2003; Carlson et al., 2009). These results suggest that neuroplasticity is present in older adults, which can be the neurobiological substrate of CR.

## Mechanistic support from preclinical models of Alzheimer's disease

While much of the clinical evidence supporting cognitive interventions in AD is indirect or based on short-term cognitive and neuroimaging outcomes, emerging preclinical studies provide important mechanistic support for the proposed neuroplasticity-based framework. In a recent study using the TgF344-AD rat model, Casanova-Pagola et al. (2025) demonstrated that early and sustained cognitive stimulation preserved functional connectivity, enhanced synaptic plasticity markers, and modulated neuroinflammatory processes, despite ongoing amyloid pathology. Complementary findings from other animal studies indicate that environmental enrichment and cognitive engagement enhance synaptic plasticity markers, promote neurogenesis, and modulate neuroinflammatory pathways relevant to AD progression (Martinez-Coria et al., 2015; Burnyasheva et al., 2020; Yang et al., 2022). Although these studies primarily assessed molecular, cellular, and regional outcomes rather than large-scale network connectivity, their findings support the biological plausibility that cognitive stimulation may contribute to neural resilience in the presence of pathology.

## Cognitive stimulation therapy as an illustrative example of non-pharmacological intervention

Non-pharmacological interventions of AD can be categorized into cognitive training (CT), cognitive stimulation (CS), cognitive rehabilitation, and combined interventions such as combination of cognitive interventions with physical exercise (Xiang and Zhang, 2024). CT involves a standardized task with different difficulty levels, aiming at improving specific cognitive domains. CS involves a wide range of group-oriented social events, aiming at generally improving cognitive function and behavior. Cognitive rehabilitation is an individualized method, aiming at achieving optimal levels of physical, psychological, and social functioning. A systematic review of 39 randomized control trials showed a moderate and statistically significant improvement in global cognition among individuals with AD for all types of cognitive interventions compared to control (Xiang and Zhang, 2024). Combined interventions had the highest surface under the cumulative ranking curve value, followed by CT, CS, and cognitive rehabilitation. Significant effects of cognitive interventions were found on working memory, verbal memory, verbal fluency, confrontation naming, attention, neuropsychiatric symptoms, basic activities of daily living, and quality of life (Xiang and Zhang, 2024).

One promising strategy for enhancing CR is fostering social participation. As noted by Sommerlad et al. (2023) social engagement may enhance CR because social interactions are cognitively demanding and involve multiple cognitive domains. The complexity of social interactions, including navigating various situations, communicating, and problem-solving, may have been a driving force in the evolutionary development of the brain. Therefore, social participation encouraging spontaneous expression of individual capacities and supporting creative problem-solving in a complex real-world situation may be effective to enhance CR.

Cognitive stimulation therapy (CST) is the only manualized international non-pharmacological intervention method recommended in clinical guidelines for people with mild to moderate Alzheimer's disease dementia. It consists of various group activities and discussions to enhance cognitive and social functioning (Woods et al., 2012). Recently, the neural, cognitive, and behavioral effects of CST were investigated in AD patients (Behfar et al., 2023). CST clearly improved cognitive functions that were measured by Mini-Mental State Examination (MMSE), the Alzheimer's Disease Assessment Scale, cognitive subsection (ADAS-cog) as well as behavioral and psychological symptoms of dementia (BPSD) scores. The improvement in cognitive functions was associated with years of education as a proxy of CR. It was further shown that cognitive improvements were associated with an up-regulated functional connectivity between the left posterior hippocampus and the trunk of the left postcentral gyrus, measured by MRI (Behfar et al., 2023). CST may thus improve cognitive function associated with CR by strengthening neural functional connectivity in the AD patients' brain.

## Discussion

The up-regulation of neural functional connectivity by CST in AD patients confirms that neuroplasticity is present in AD patients. Non-pharmacological approaches may thus increase CR and mitigate cognitive decline by stimulating neuroplasticity in the brain (Figure 1). On the other hand, pharmacological approaches using amyloid-β antibody aim to delay cognitive decline by reducing AD pathology in the brain (Figure 1). It can be summarized that pharmacological approach is an attempt to reverse the normal aging process of amyloid-β accumulation that may increase until death without treatment. On the other hand, non-pharmacological approaches represent a complementary and biologically plausible strategy that utilizes residual neuroplastic capacity in the aging brain. Mitigating or delaying cognitive decline may require enhancing neuroplasticity through non-pharmacological approaches, in addition to reducing pathological burden through pharmacological interventions (Figure 1).

In summary, this Opinion reframes non-pharmacological interventions not as adjunctive or symptomatic treatments, but as network-level strategies that enhance cognitive reserve through neuroplasticity. By integrating human neuroimaging findings with emerging mechanistic evidence from animal models, we propose a biologically grounded framework in which cognitive stimulation increases resilience to Alzheimer's disease pathology, thereby modifying the clinical expression of the disease rather than fully reversing pathology itself.
